# Coding-complete genome of human cytomegalovirus extracted from a nasopharyngeal swab in Brazil

**DOI:** 10.1128/mra.00985-25

**Published:** 2026-01-13

**Authors:** Amanda Mendes Silva Cruz, Edivaldo Costa Sousa, Luana Soares Barbagelata, Wanderley Dias das Chagas, Agatha Monike Silva Nunes, Miriam Teresinha Furlam Prando Livorati, Walquiria Aparecida Ferreira de Almeida, João Farias da Trindade, Andreia Santos Costa, Fernando Neto Tavares, Maísa Silva de Sousa, Mirleide Cordeiro dos Santos

**Affiliations:** 1 Laboratory of Respiratory Viruses, Section of Virology, Evandro Chagas Institute, Ministry of Health 89124, Ananindeua, Pará, Brazil; 2 Graduate Program in Pathology of Tropical Diseases, Federal University of Pará 37871, Belém, Brazil; 3 Labepileish, Section of Parasitology, Evandro Chagas Institute, Ministry of Health, Ananindeua, Pará, Brazil; 4 General Coordination of Public Health Laboratories, Brazilian Ministry of Health, Brasília, Brazil; 5 Secretariat of Health and Environmental Surveillance, Brazilian Ministry of Health, Brasília, Brazil; 6 Secretariat for Health of Amapá State, Macapá, Brazil; 7 Central Public Health Laboratory of the Amapá State, Brazilian Ministry of Health, Macapá, Brazil; Katholieke Universiteit Leuven, Leuven, Belgium

**Keywords:** *Cytomegalovirus humanbeta5*, Brazil

## Abstract

We report the coding-complete genome sequence of *Cytomegalovirus humanbeta5* (BR/2023-012844/IEC/NS/2023), recovered from a nasopharyngeal swab collected from a 5-month-old infant with severe acute respiratory syndrome during a respiratory syncytial virus outbreak in Brazil.

## ANNOUNCEMENT

Human cytomegalovirus (HCMV) is a member of the family *Herpesviridae*, subfamily *Betaherpesvirinae*, and genus *Cytomegalovirus* (1). Its genome consists of a double-stranded DNA molecule of >200 kbp (2). HCMV genomes are most commonly recovered from blood, urine, and saliva, particularly in congenital infections and in immunocompromised individuals (3–7). Recovering viral genomes from upper respiratory tract samples, such as nasopharyngeal swabs, is uncommon because this anatomical site is not routinely examined for HCMV in clinical or surveillance settings. However, obtaining the viral genome from the nasopharynx may provide key insights into viral shedding, reactivation, and coinfection dynamics. Shotgun metagenomics is well suited for recovering viral genomes directly from clinical samples (3). Notably, as of 1 September 2025, only partial HCMV genome sequences from Brazil were available in GenBank (8).

The nasopharyngeal swab analyzed in this study (2023-012844/IEC) was obtained from a 5-month-old infant hospitalized in an intensive care unit in Macapá, Amapá, located in the Brazilian Amazon. The GPS coordinates for sample collection were 0.0388900 and −51.0663900. The sample was collected in April 2023 by the Health Surveillance Secretariat during a respiratory syncytial virus (RSV) outbreak in Brazil. Viral DNA was extracted using the Extracta Kit – Pathogen DNA and RNA MDx, which employs magnetic microparticle technology, following the manufacturer’s instructions. Library preparation and sequencing were performed at the Respiratory Virus Laboratory of the Evandro Chagas Institute. Libraries were constructed using the Illumina Nextera XT DNA Library Preparation Kit (Illumina, San Diego, CA, USA) and sequenced on the Illumina NextSeq 550 System with 2 × 150 bp paired-end reads, generating 39,712,796 read pairs. Quality filtering of reads in FASTQ format was performed using fastp v.0.23.1 (9), and *de novo* assembly was conducted using MEGAHIT v.1.2.9 (10) (Table 1). Taxonomic classification of contigs was carried out using Kraken2 v.2.1.0 (11) with the Standard-16 database, which identified multiple contigs assigned to *Cytomegalovirus*. Classification outputs were visualized using the Pavian R package v.1.2 (12). Manual review and open reading frame prediction were performed in Geneious Prime (13), where multiple overlapping contigs were identified. These contigs, supported by read mapping and alignment with MAFFT (14), allowed reconstruction of a coding-complete genome relative to the reference strain (GenBank accession number PP803491). Each contig was additionally confirmed by BLASTn (15).

**TABLE 1:** Sequencing and genome metrics

Metric	Data for BR/2023-012844/IEC/NS/2023
No. of raw reads	39,712,796
No. of reads after trimming	36,229,206
Average read length (bp)	158
Total no. of contigs	912,240
No. of contigs related to *Cytomegalovirus humanbeta5*	16
Genome size (bp)	231,043
GC content (%)	57.5
Coverage (×)	801.4
N50	466
GenBank accession no.	PX246278

The 2023-012844_CMV/IEC genome exhibited a typical HCMV genomic organization, with a length of 231,043 bp and 178 predicted genes. The mean read coverage was 801.4×, and the GC content (57.5%) was consistent with other *Cytomegalovirus humanbeta5* genomes available in GenBank. The genome displayed high nucleotide identity (>98%) with related HCMV strains available in GenBank (KJ361950; KJ361951; KJ426589; KT726951; KY490088; MT044485), demonstrating the genetic similarity of the 2023-012844/IEC strain to previously characterized isolates. The shotgun metagenomic approach provided evidence of HCMV genomic material in the nasopharynx of an RSV-positive infant with severe acute respiratory syndrome. As RSV infection can modulate host immune responses (16), further investigations are warranted to elucidate viral coinfections, HCMV reactivation, and the potential impact of congenital HCMV infection.

## Data Availability

The whole genome sequencing project of sample BR/2023-012844/IEC/NS/2023 was deposited in GenBank under accession number PX246278. The accession number for the raw shotgun metagenomic sequencing reads in the NCBI Sequence Read Archive is SRR35176174. This data set is associated with BioProject accession number PRJNA1312120 and BioSample accession number SAMN50854041 (2023-012844/IEC).
